# Cultural differences in wine conceptualization among consumers in France, Portugal and South Africa

**DOI:** 10.1038/s41598-024-66636-3

**Published:** 2024-07-10

**Authors:** Samantha Fairbairn, Jeanne Brand, Antonio Silva Ferreira, Dominique Valentin, Florian Bauer

**Affiliations:** 1 South African Grape and Wine Research Institute, Stellenbosch University, Stellenbosch, South Africa; 2 Escola Superior de Biotecnologia, Universidade Católica Portuguesa, Rua Dr. António Bernardino de Almeida, 4200-072 Porto, Portugal; 3 Centre des Sciences du Goût et de l’alimentation, AgroSup Dijon, CNRS, INRA, Université Bourgogne Franche-Comté, F-21000 Dijon, France

**Keywords:** Wine aroma, Consumer, Culture, Conceptualisation, Human behaviour, Psychology

## Abstract

Consumers’ mental pictures of wine are multifaceted and are shaped by their sensory (taste, smell, visual, sensation) perceptions, in addition to emotional, cultural and extrinsic (brand, price, and awards) influences. This study explores whether consumers from three different wine cultures share mental representations of three wine concepts. Through an online survey, French, Portuguese, and South African wine consumers described their conceptualizations of *Wine, Red wine* and *White wine* aroma. Given these nations’ rich winemaking traditions and diverse wine styles, differences in consumer perspectives were likely to emerge. The findings demonstrate that, regardless of cultural background, the broad concept of *Wine* aligns with the more specific *Red* and *White wine* conceptualizations, although the latter concepts diverge from each other. Notably, cultural contexts significantly influence participants’ representations of *Red Wine*, with particularly marked contrasts between the South African and French respondents. This suggests that like experts, wine consumers have also built representations of wine through semantic memory. This cross-cultural analysis of consumer interpretations of wine concepts holds the potential for refining marketing strategies to overcome cultural barriers in wine purchasing behaviour.

## Introduction

Wine is a grape-derived alcoholic beverage with a long history and deep cultural resonance as evidenced by its prominent role in literature and visual representations since antiquity^1^. In modern times, it can also claim to be the most sensorially analysed and described food-item with a very large descriptive vocabulary^2^. Food and wine are often consumed in social contexts which also impacts our perception of these products^3,4^. It is within these contexts, or cultures, that we develop and share our beliefs, values and wine consumption rituals and practices^3^. Research indicates that wine consumers attribute significant importance to the context of wine consumption, encompassing factors such as the occasion being commemorated, the individuals present, and the pairing with food^5,6^. Consequently, national- and cross-cultural research has received much interest, and the latter was recently reviewed by Rodrigues and Parr^7^.

Examining the global wine landscape reveals that Portugal, France and South Africa are among the top 16 in terms of wine production, exportation and consumption based on the International Organisation of Vine and Wine’s (OIV) 2020 data^8^. However, only the European countries are among the top ten wine importers with South Africa lagging far behind as the 83^rd^ largest importer. Consequently, South Africans mostly consume their domestic product, whereas French and Portuguese consumers have exposure to international wines.

Moreover, within Europe, the significance of *terroir* (place of origin) is pronounced, often standing as a representation of national pride. This reverence finds expression in wine marketing strategies, labelling, and descriptions^9,10^. However, in South Africa, as in much of the new world, the emphasis of producers and marketers shifts to more technical aspects such as grape cultivar and method of production, setting their wine perspective apart from their European counterparts^9^. This cultural distinction came to the fore in a comparative analysis involving Chenin blanc experts in South Africa and France. South Africans predominantly considered the wine’s inherent characteristics. Conversely, the French placed greater emphasis on the wine’s origin and the consumption context (meal and aperitif). This divergence underscores the varied perspectives between these two cultures^9^. Portuguese and South African wine consumers have not been studied as extensively as French consumers. Nonetheless, research has shown that Portuguese and South African consumers, like their French counterparts, pair wine with meals^5,6,9,11^. Portuguese consumers associate drinking wine with feeling calm and comforted^5^, the French with conviviality^12^ and South Africans drink wine to feel relaxed and confident as well as an outward display of sophistication and status (when drinking red wine in particular)^5^. This association of wine with success is a trait also seen among Asian wine consumers^11^.

A recent review by Barbe et al.^13^ outlined a three-step process to describe the sensory space of wine: The first is to determine whether the sensory concept exists and is shared on a purely conceptual basis, this is followed by a perceptual evaluation of the product using categorization and descriptive tasks. In a recent study, our research group explored *Saccharomyces cerevisiae’s* ability to create a global wine-like aroma profile, by fermenting a minimal defined medium without any varietal aroma precursors^14^. Alterations in this medium ultimately resulted in the perception of a wine-like character using rating, categorization, and descriptive tasks. Interestingly, the attributes elicited were ones associated with white wine. This raised the question of whether this global wine character exists and whether it is shared at a conceptual level. Recently, wine research has included the purely cognitive description of wine, primarily by experts to describe various wines styles or wine concepts. This approach has been used to understand the meaning of wine complexity^15^, to profile the typicality of Portuguese white wines^16^, of wines within the Beaujolais region^17^ and to characterize the green character of red wine^18^. This approach removes biases linked to the selection of suitable wines at adequate numbers for evaluation.

This study aimed to first examine whether people have different mental representations of red and white wine compared to a “global wine concept” and secondly to compare the degree to which culture impacts these representations among South African, French, and Portuguese wine consumers. Consumers and experts approach describing wine differently. Consumers describe the inherent characteristics of wine (which we are interested in), whereas experts usually describe it by comparing it to an idealised prototype of a specific wine style often using technical winemaking terms^15,19^.

## Materials and method

### Participants

Respondents were South African, French, or Portuguese wine consumers without any formal wine-related training, and who consume wine at least once a month. They were recruited via email among the staff and postgraduate students at Stellenbosch University, the University of Burgundy, and the Catholic University of Portugal at Porto. As is often the case in cross-cultural studies, the convenience sampling method used has some limitations including the sample of participants not being representative of the whole country’s population, but rather a regional self-selected sub-population.

South Africa, France and Portugal were selected for comparison based on data shown in Table 1. These countries are among the top sixteen wine producing and consuming countries, although there are notable disparities in their wine consumption. South Africans consume significantly less wine per capita than their European counterparts, however our inclusion criteria mitigate this disparity by using the frequency of consumption of respondents. This study also aims to contrast the cultural dimensions of wine mental representation between a ‘New World’ (South Africa) and two ‘Old World’ countries with very different wine consumption and appreciation histories (France and Portugal), including significant varietal differences. The data may also reflect the effects of a more global wine exposure in France and Portugal compared to South Africa’s insular market.

**Table 1:** A summary of the wine production, consumption, import and export data and the corresponding world ranking in terms of wine volume (OIV, 2020) of South Africa, France, and Portugal used as criteria for selection for participation.

New world		Old world	
1000 hL	South Africa	France	Portugal
Production	10 385 (7th)	46 673 (2nd)	6 418 (11th)
Consumption	3 138 (16th)	24 361 (2nd)	4 374 (12th)
Per capita (L)	7.5	45.8	49.1
Exports	3 620 (8th)	14 085 (3rd)	3 151 (10th)
Imports	44 (83rd)	6 561 (4th)	2 745 (10th)

The socio-demographic data collected were age, sex, nationality, and education. For the South African cohort, 186 responses (69% female, 30% male, 1% undisclosed) met the inclusion criteria compared to 46 French (57% female, 41% male, 2% undisclosed) and 62 Portuguese (55% female, 45% male) respondents (Table 2). The difference in number of participants between countries can be explained by the fact that data collection was carried out during COVID and at this time the availability of staff and students differed among the three universities. To have a more balanced dataset, the data of 60 South African (72% female, 28% male) consumers were randomly selected and used in subsequent analyses (Table 2). Multiple factor analysis (MFA, performed using XLSTAT® software, Addinsoft, 2023.) comparing the full South African dataset to this subset of 60 random consumers (Supplementary figure S1) showed that they are similar.

**Table 2:** Consumer socio-demographic data.

	South Africa (n = 60)	France (n = 46)	Portugal (n = 62)
**Sex**			
Female	43	26	34
Male	17	19	28
Undisclosed	0	1	0
**Age** (average ± standard deviation)			
Baby Boomer	62.63 ± 4.66	61.5 ± 5.36	61.00 ± 3.00
Gen X	49.50 ± 4.37	49.65 ± 3.22	50.02 ± 3.26
Gen Y	31.27 ± 5.20	31.60 ± 5.27	31.56 ± 4.29
Gen Z	23.25 ± 0.89	22.46 ± 1.13	21.50 ± 2.12
**Education**			
High School or less	1	5	2
Tertiary education	59	41	60
**Employment**			
Employed	43	26	50
Student	11	14	5
Retired	2	2	1
Unemployed	1	0	3
Other	3	4	3

Consumers’ wine consumption data with respect to wine training, consumption frequency, involvement (wine interest) and regular consumption preference were also collected (Table 3). To determine their wine interest or involvement, participants rated the degree to which certain word pairs (important/unimportant, interesting/boring, relevant/irrelevant, exciting/unexciting, means a lot/ means nothing, appealing/unappealing, fascinating/mundane, valuable/worthless, involving/uninvolving and, needed/not needed) described their relationship with wine using a 7-point scale. The participant’s context-free wine involvement was estimated by averaging these scores^20^ (Table 3) where seven indicates a high wine involvement and one low involvement. An overview of how respondents rated these questions is summarised in Supplementary Figure S2.

**Table 3:** The frequency data summarizes the respondent’s wine consumption habits, specifically which wine types they regularly consume and how often, as well as their wine involvement.

	South Africa (n = 60)	France (n = 46)	Portugal (n = 62)
**Regularly consumes**			
Red	48 (80%)	41 (89%)	48 (77%)
White	33 (55%)	41 (89%)	46 (74%)
Rosé	8 (13%)	16 (35%)	18 (29%)
Sweet	6 (10%)	8 (17%)	6 (10%)
Sparkling	18 (30%)	24 (52%)	20 (32%)
**Consumption frequency**			
Daily	5 (8%)	4 (9%)	10 (16%)
Several times a week	18 (30%)	14 (30%)	22 (35%)
Once a week	15 (25%)	7 (15%)	9 (15%)
Several times a month	17 (28%)	19 (41%)	15 (24%)
Once a month	5 (8%)	2 (4%)	6 (10%)
**Wine involvement score**			
Very low: < 2	1 (2%)	1 (2%)	0 (0%)
Low: 2–3	0 (0%)	2 (4%)	3 (5%)
Low: 3–4	4 (7%)	3 (7%)	8 (13%)
Neutral: 4–5	14 (23%)	19 (41%)	13 (21%)
High: 5–6	25 (42%)	10 (22%)	25 (40%)
Very high: > 6	16 (27%)	11 (24%)	13 (21%)

The sex distribution was similar for the French (57% female, 41% male, 2% undisclosed) and the Portuguese (55% female, 45% male) cohorts and differed from the South African participants (72% female, 28% male). Additionally, most of the consumers had a tertiary education and were either employed or students.

This simple wine involvement test is used as a proxy for wine interest and knowledge, the premise being that the more interested (involved) you are in a subject the more familiar and knowledgeable you would be^20,21^. More than 80% of the participants, in each cultural cohort, had a neutral to high wine involvement (Table 3), as shown by their averaged ratings. Wine involvement is more complex and nuanced than suggested here, as it is impacted by several factors including the consumers’ context (situation)^22^.

The participants generally consumed red wine (South Africa 80%, France, 89% and Portugal 77%) and white wine (South Africa 55%, France, 89% and Portugal 74%), with South Africans appearing to consume red wine more regularly than white wine, whereas their European counterparts consume them at similar frequencies.

### Ethical approval

This research was approved by the Ethics Committee of Stellenbosch University (10,095) and conducted in accordance with their guidelines. Informed consent was obtained from all participants.

### Procedure

All responses were collected using the Compusense^®^ online platform (Compusense Inc., Guelph, Canada). As a warm-up exercise, participants were asked to describe a car. A minimum of three descriptors was required and a maximum of 16 permitted. They were then tasked to describe wine aroma. The question was posed as follows: “Imagine trying to make an alien understand the differences between different drinks. Which descriptors would you use to describe the smell of *Wine*?”. Participants then described *Red* and *White wine* concepts. The order of the latter two concepts was randomized.

### Data analysis

#### Text analysis

All analyses were carried out in the original language of the participants. Spelling errors were corrected, and the descriptors were subjected to lemmatization which reduces words to their root (e.g., changing berries to berry). The frequency of occurrence of lemmatized descriptors (for each concept and country) was computed and tabulated in frequency tables with descriptors as row and concept/country as columns.

Descriptors with the same semantic meaning were then grouped (categorization) whilst retaining as much nuance as possible to ensure that cultural differences are retained (e.g., floral, flowers and blossoms were characterised as floral). This was done independently by three researchers in the original language following the same grouping rules in all three datasets. The French and Portuguese final descriptors were translated into English by a native French or Portuguese speaker for further analyses. The frequency of occurrence of each descriptor for each concept in each country was then tabulated and summed as described above to create contingency tables.

#### Aroma attribute specificity

The differences in the aroma descriptor specificity were evaluated across and within wine concepts and countries. The characterised descriptors were classified as generic (e.g., fruity), intermediate (e.g., tropical fruit) and specific (e.g., pineapple) largely using the aroma wheel as a guide^23,24^. The frequency with which attributes, at each specificity level, were used to describe the concepts, in each country, was calculated. Pearson’s correlation coefficients and Chi (χ^2^) square independence tests were calculated between all pairs of concepts (*Wine* vs *White wine*; *wine* vs *Red wine*; *White wine* vs *Red wine*) within each country and across countries (South Africa vs France; South Africa vs Portugal; France vs Portugal) for each concept. Furthermore, the relationship between the wine concepts, wine cultures and characterised attributes for the generic, intermediate, and specific attributes were illustrated using correspondence analysis (Supplementary figure S3). All analyses were performed using XLSTAT^®^ software.

#### Relationships between all descriptors elicited and the wine concept and or culture

To evaluate the relationship between all the characterised attributes elicited for the three concepts in the three countries, once again Pearson’s correlation coefficients and Chi-square tests were conducted as discussed. To visualize the relationship between the words used to describe the three wine concepts correspondence analyses were performed. The first set of three CAs (one per country) compared the descriptors associated with the three wine concepts within each country. The second set (one per wine concept) compared the descriptors associated with a wine concept across the three countries. All CAs were performed on descriptors mentioned by at least 10% of the participants in at least one wine concept or country depending on the context, using XLSTAT^®^ software.

## Results

### Aroma attribute specificity

The aroma attributes were grouped based on their degree of information specificity as generic (fruity), intermediate (tropical or exotic fruit), or specific (pineapple) using an aroma wheel^23^. Table 4 shows the frequency with which words in these three levels of descriptor specificity were associated with the *Wine, White wine* and *Red wine* concepts in the three countries evaluated.

**Table 4:** The percentage of wine aroma attributes classified as generic, intermediate, or specific based on the aroma wheel used to describe the wine concepts.

		Wine (%)	Red Wine (%)	White Wine (%)	Characterised attributes
South Africa	Specific	14	16	32	barrel (RW, W), butter (WW), chocolate (RW), cork (RW, W), dusty (RW, W), grape (All), grass (WW, W), musty (RW, W), smoke (RW, W)
	Intermediate	12	26	45	green (WW), fresh (All), berry (RW, W), black_fruit (RW), red_fruit (RW), tropical/exotic_fruit (WW, W), white_fruit (WW), citrus (All), crisp (WW)
	Generic	74	58	43	fruity (All), undergrowth (RW, W), animal (RW, W), nutty (RW, W), spicy (All), floral (WW, W), mineral (RW, WW), wooded (All)
France	Specific	10	12	6	barrel (tonneau-RW, W), bell_pepper (légume-RW), butter (beurré-RW, WW), grape (raisin-All), iron (fer-RW, W), smoke (fumée-WW, W), vanilla (vanillé-All)
	Intermediate	5	24	23	citrus (agrume-WW), fresh (frais-RW, WW), green (vert-WW, W), red_fruit (fruit_rouge-RW, W), stone fruit (fruit_à_noyaux-WW), tropical/exotic_fruit (fruit_exotique-WW)
	Generic	86	64	71	animal (animal-RW, W), floral (floral-All), fruity (fruité-All), mineral (mineral-WW, W), nut (fruit_sec-WW, W), spicy (épice-All), undergrowth (sous-bois-All), vegetal (végétal-All), wooded (boisé-All)
Portugal	Specific	3	6	3	grape (uva-All), walnuts (nozes-RW)
	Intermediate	13	21	41	black_fruit (fruta preta-RW, W), citrus (citrico-WW), dark_fruit (frutos pretos-RW, W), fresh (fresco-WW, W), red_fruit (frutos vermelha-All), tropical/exotic_fruit (frutos tropicais-WW), white fruit (Frutos brancos-WW, W)
	Generic	84	73	56	floral (floral-All), fruity (frutado-All), mineral (mineral-WW), nutty (frutos_secos-RW), spicy (especiarias-All), undergrowth (vegetação rasteira-RW), wooded (arborizada-All)

The original language is shown in the parentheses as well as which concept was described: *Red wine* (RW), *White wine* (WW), *Wine* (W) or all three concepts (All).

In all countries, when the wine concept evaluated is more precise, either *White wine* or *Red wine*, consumers generally provided more informative (intermediate and specific) and fewer generic attributes (Table 4). For the French consumers, 86% of the attributes used to describe the global *Wine* concept were generic, compared to 64% and 71% for the *Red wine* and *White wine*, respectively. Interestingly, the data trends suggest that South African consumers were more detailed (specific) in their descriptions than their European counterparts, as shown by the comparatively lower frequencies for the generic descriptors.

At the generic description level, the data show that the foundation of all three wine concepts, regardless of culture, is fruity, spicy and wood whereas other generic descriptors are context specific (Table 4). Minerality was associated with *White wine* in all three countries, but it was also associated with the global *Wine* concept by French and the *Red wine* concept by South African consumers. Some other context differences include French (green, vegetal) and South African (grass, green) consumers highlighting the presence of vegetal and green aromas which are generally absent from the Portuguese data (Table 4). French and South African consumers also cited animal related descriptors, which are entirely absent from the Portuguese dataset.

When comparing the relationship between the different wine concepts, within a culture, the correlation data suggests that the global *Wine* concept generally correlates with the *Red* or *White wine* concepts for the generic attributes (Table 5, Supplementary figure S3). However, the Chi-square test of independence analyses show that the relationship between the *Wine* and *White wine* concepts may not be significant in the French cohort (χ^2^ = 12.39, df = 8, *p* = 0.13). Additionally, with the increase in the specificity of the descriptor any correlation is generally lost, and cultural differences emerge (Supplementary figure S3).

**Table 5:** Relationship (Pearson’s correlation and Chi-square tests) between the generic, intermediate, and specific attributes used to describe the wine concepts within cultures.

			Pearson’s correlation coefficient	Chi-squared (χ^2^)	DF	*p*-value
South Africa	Specific	Red Wine-White Wine	− 0.396	26.15	8.00	**< 0.001**
		Red Wine-Wine	0.021	8.82	7.00	0.27
		White Wine-Wine	0.506	11.98	7.00	0.10
	Intermediate	Red Wine-White Wine	− 0.361	72.56	8.00	**< 0.001**
		Red Wine-Wine	0.752	10.30	5.00	0.07
		White Wine-Wine	− 0.122	36.28	6.00	**< 0.001**
	Generic	Red Wine-White Wine	0.326	55.35	7.00	**< 0.001**
		Red Wine-Wine	0.606	23.31	7.00	**< 0.001**
		White Wine-Wine	0.929	18.73	7.00	**0.01**
France	Specific	Red Wine-White Wine	0.539	4.58	6.00	0.60
		Red Wine-Wine	0.281	4.96	6.00	0.55
		White Wine-Wine	− 0.556	3.11	5.00	0.68
	Intermediate	Red Wine-White Wine	− 0.382	33.32	5.00	**< 0.001**
		Red Wine-Wine	0.904	5.25	2.00	0.07
		White Wine-Wine	− 0.556	20.04	5.00	**< 0.001**
	Generic	Red Wine-White Wine	0.417	43.40	8.00	**< 0.001**
		Red Wine-Wine	0.769	20.55	8.00	**0.01**
		White Wine-Wine	0.868	12.39	8.00	0.13
Portugal	Specific	Red Wine-White Wine	1.000	1.60	1.00	0.21
		Red Wine-Wine	1.000	1.12	1.00	0.29
		White Wine-Wine	*	*	*	*
	Intermediate	Red Wine-White Wine	− 0.367	54.40	6.00	**< 0.001**
		Red Wine-Wine	− 0.414	17.71	4.00	**< 0.001**
		White Wine-Wine	0.858	13.31	6.00	**0.04**
	Generic	Red Wine-White Wine	0.336	44.24	6.00	**< 0.001**
		Red Wine-Wine	0.655	18.33	5.00	**< 0.001**
		White Wine-Wine	0.850	15.28	4.00	**< 0.001**

*Too few attributes to analyse.

Numbers in bold denote *p*-values smaller than 0.05.

When comparing the wine concepts across cultures, the generic attributes correlated, however, this correlation was once again not necessarily significant (Table 6), as shown by the Chi-square independence test. Generally, the French and Portuguese generic wine concepts correlated well—*Wine*: χ^2^ = 17.46, df = 8, *p* = 0.03, *Red wine*: χ^2^ = 28.36, df = 7, *p* = 0.00, and *White wine*: χ^2^ = 16.44, df = 7, *p* = 0.02.

**Table 6:** Relationship (Pearson’s correlation and Chi-square tests) between the generic, intermediate, and specific attributes used to describe the wine concepts across culture.

			Pearson’s correlation coefficient	Chi-squared (χ^2^)	DF	*p*-value
Red Wine	Specific	South Africa-Portugal	− 0.257	15.17	7.00	**0.03**
		France-Portugal	0.359	7.47	6.00	0.28
		South Africa-France	− 0.340	18.49	10.00	0.05
	Intermediate	South Africa-Portugal	− 0.293	29.45	5.00	**< 0.001**
		France-Portugal	0.838	9.63	3.00	**0.02**
		South Africa-France	− 0.266	32.73	4.00	**< 0.001**
	Generic	South Africa-Portugal	0.820	21.67	7.00	**< 0.001**
		France-Portugal	0.602	28.36	7.00	**< 0.001**
		South Africa-France	0.740	15.72	8.00	0.05
White Wine	Specific	South Africa-Portugal	− 0.381	6.68	2.00	**0.04**
		France-Portugal	1.000	2.25	3.00	0.52
		South Africa-France	− 0.358	9.68	4.00	**0.05**
	Intermediate	South Africa-Portugal	0.858	16.61	6.00	**0.01**
		France-Portugal	0.960	10.45	6.00	0.11
		South Africa-France	0.751	14.67	6.00	**0.02**
	Generic	South Africa-Portugal	0.934	4.42	4.00	0.35
		France-Portugal	0.906	16.44	7.00	**0.02**
		South Africa-France	0.864	19.99	7.00	**0.01**
Wine	Specific	South Africa-Portugal	0.471	4.62	6.00	0.59
		France-Portugal	0.875	2.50	4.00	0.64
		South Africa-France	0.426	7.43	8.00	0.49
	Intermediate	South Africa-Portugal	-0.033	15.78	7.00	**0.03**
		France-Portugal	-0.279	11.16	5.00	0.05
		South Africa-France	-0.543	15.00	5.00	**0.01**
	Generic	South Africa-Portugal	0.965	10.40	6.00	0.11
		France-Portugal	0.969	17.46	8.00	**0.03**
		South Africa-France	0.936	16.40	8.00	**0.04**

Numbers in bold denote *p*-values lower than 0.05.

The aroma-only data shows that the wine conceptualizations of Portuguese, French and South African wine consumers are well aligned at the generic level (Supplementary figure S3), but as the specificity of the attribute increases nuances are more apparent.

### Relationships between all descriptors elicited and the wine concept and or culture

Chi-square independence test was used to evaluate whether a relationship exists between two categorical variables, firstly comparing the wine concepts within (Table 7) and then across cultures using all the elicited categorized data. The nature of these relationships was further described using Pearson’s correlation coefficients.

**Table 7:** Relationship (Pearson's correlation and Chi-square test) between wine concepts within and across cultures.

		Pearson’s correlation	Chi-square independence test (χ^2^)	Degrees of freedom	*p*-value
Wine	South Africa-France	0.77	166.72	80	**< 0.0001**
	South Africa-Portugal	0.79	167.56	87	**< 0.0001**
	Portugal-France	0.83	142.20	72	**< 0.0001**
Red Wine	South Africa-France	0.37	201.97	79	**< 0.0001**
	South Africa-Portugal	0.57	180.57	89	**< 0.0001**
	Portugal-France	0.52	167.29	78	**< 0.0001**
White Wine	South Africa-France	0.75	123.26	55	**< 0.0001**
	South Africa-Portugal	0.86	114.31	56	**< 0.0001**
	Portugal-France	0.79	101.56	55	**< 0.0001**
South Africa	Red Wine-White Wine	0.28	263.69	72	**< 0.0001**
	Red Wine-Wine	0.65	114.62	70	**0.001**
	White Wine-Wine	0.69	155.01	64	**< 0.0001**
France	Red Wine -White Wine	0.44	134.81	47	**< 0.0001**
	Red Wine-Wine	0.72	69.66	46	**0.014**
	White Wine -Wine	0.79	84.58	47	**0.001**
Portugal	Red Wine -White Wine	0.31	208.41	65	**< 0.0001**
	Red Wine -Wine	0.74	86.09	62	**0.023**
	White Wine -Wine	0.69	125.39	56	**< 0.0001**

Numbers in bold denote p-values lower than 0.05.

Irrespective of cultural background, the *Red wine* concept differed from the *White wine* concept (Table 7). Moreover, both the *Red wine* and *White wine* concepts demonstrated greater resemblances to the broader global *Wine* concept. Interestingly, when we compare the concepts across cultures (Table 7), the data show that *Wine* and *White wine* mental concepts were generally similar across all cultures. The *Red wine* concepts showed the poorest similarity with the South African construct more closely resembling the Portuguese (0,57) than the French (0,37). Additionally, the French *Red wine* construct more closely resembled that of the Portuguese (0,52) than the South African cohort. Indeed, these relative trends are also seen for the *Wine* and *White wine* concepts where the South African concepts were more similar to the Portuguese than the French and the French conceptualizations were more similar to the Portuguese.

**Figure 1:**
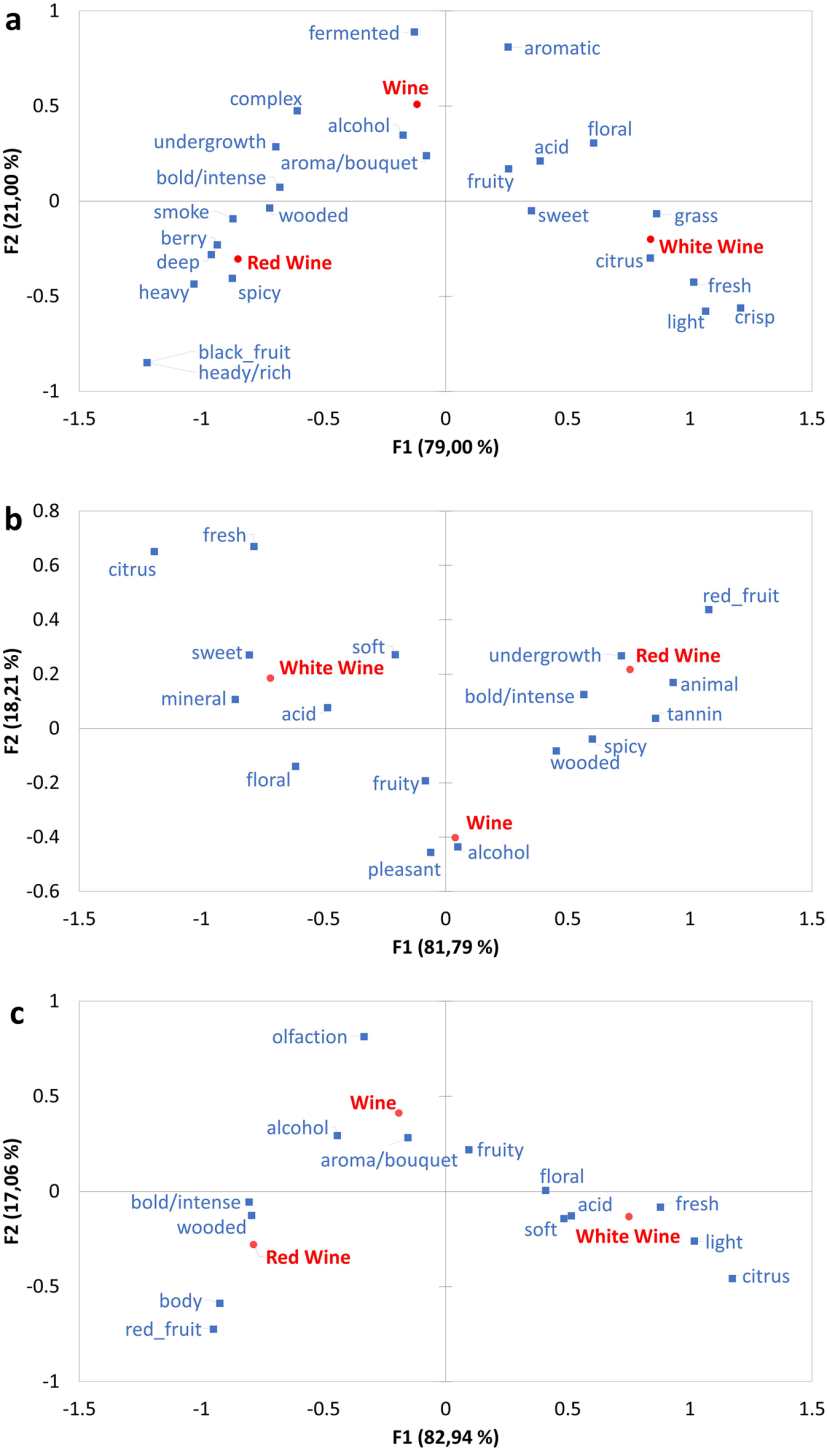
Evaluating the wine concept differences within each culture. Correspondence analysis comparing the attributes associated with *Wine*, *Red Wine* and *White* concepts by a minimum of 10% of the participants in South Africa (**a**), France and (**b**), Portugal (**c**).

**Figure 2:**
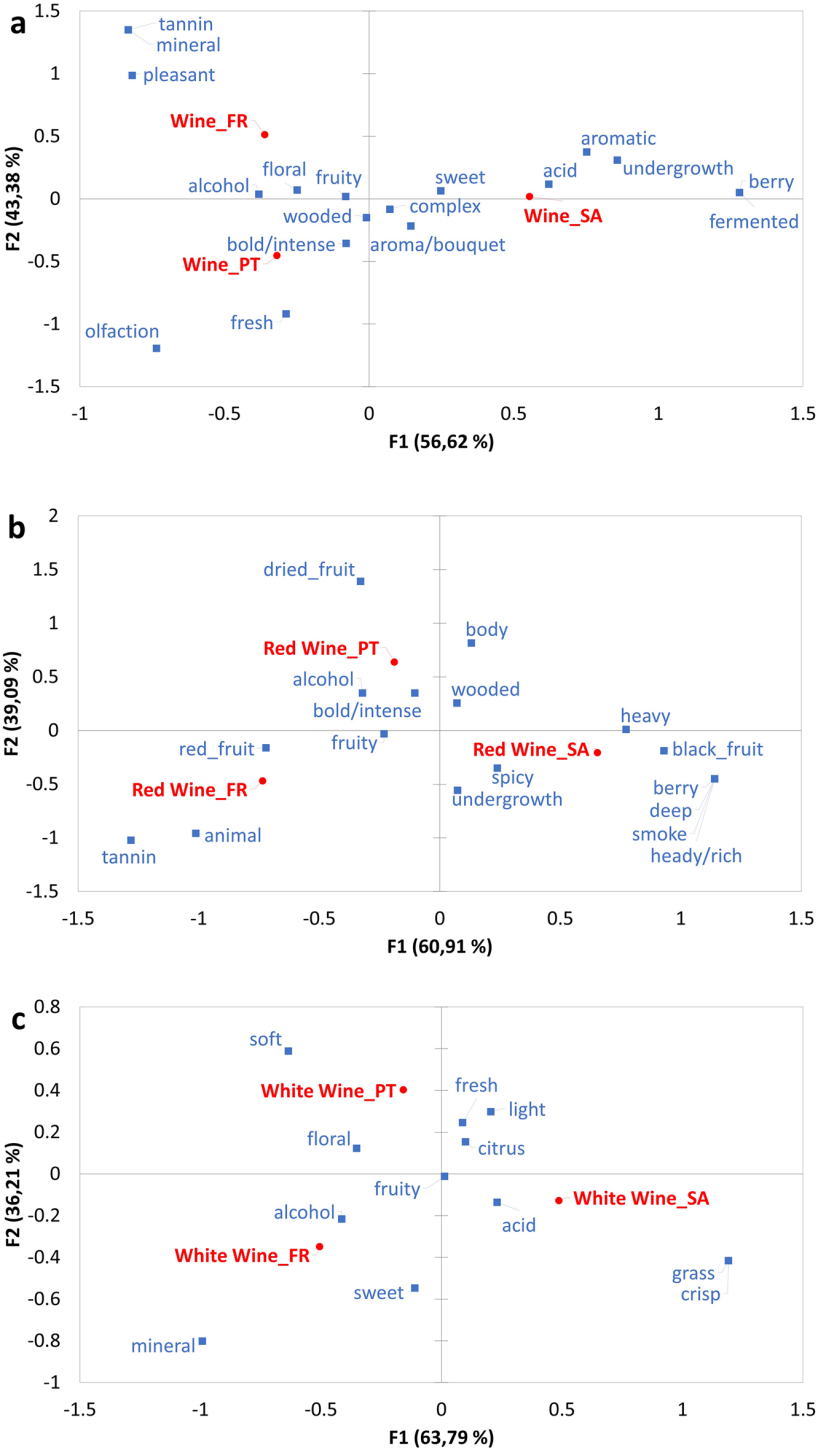
Evaluating the cultural differences for each wine concept. Correspondence analysis comparing the attributes associated with *Wine* (**a**)*, Red* (**b**) and *White Wine* concepts (**c**) when used by a minimum of 10% of the participants in at least one of the cohorts in South Africa (SA), France (FR), and Portugal (PT).

CA was conducted on the categorized data where a minimum of 10% of the participants used the attribute within (Fig. 1) or across cultures (Fig. 2). For all cultures, the first dimension describes the separation of the *Red* and *White wine* concepts (explaining between 79 and 82.9% of the variation in the data), with the general *Wine* concept, falling between them (Fig. 1). Furthermore, the second dimension describes the separation of this general concept from the two more specific concepts (explaining between 17.1 and 21.0% of the variation). This agrees with the correlation data shown in Table 7.

In all three countries, fruity was used to describe all wine concepts, although it was more frequently used to describe *Wine* and *White wine* than *Red wine*, when berry related attributes (South Africa—berry, France, and Portugal—red fruit) become more prominent. As expected, attributes often associated with red wine and white wine, were used to describe the *Red wine* and *White wine* concepts, respectively.

South Africans associated the generic *Wine* concept with acidic, alcoholic, wooded, fermented, undergrowth and aromatic attributes. The *White wine* concept was described as sweet, citrus, grassy, fresh, light, and crisp. The *Red wine* concept was associated with wooded, spicey, undergrowth, and smoky aromas (Fig. 1a).

The French participants described the *Wine* concept as pleasant, fruity, and alcoholic. The *White wine* concept was described as sweet, citrus, fresh, mineral, and soft. The *Red wine* concept was associated with wooded, spicy, undergrowth, tannin, and red fruit (Fig. 1b).

Lastly, Portuguese respondents used olfaction, alcohol, and aroma/bouquet to describe the general *Wine* concept. The *White wine* concept was described as light, citrus, fresh, acidic, and soft. The *Red wine* concept was associated with wooded and red fruit aromas (Fig. 1c).

When comparing these wine concepts across cultures, the first dimension describes the differences between the French and South African participants for the *Red* and *White wine* concepts and European and South African consumers in the case of the general *Wine* concept (Fig. 2). The second dimension generally highlights the differences between the French and Portuguese participants. Information-poor aroma descriptors such as aromatic (South Africa), aroma/bouquet (South Africa, Portugal), olfaction (Portugal), alcohol (South Africa, France, Portugal), pleasant (France), fermented (South Africa) were associated with the general *Wine* concept (Fig. 2a). All three cohorts also described the global *Wine* concept as complex, wooded and fruity.

Fruity, intensity (bold/intense) and wooded attributes were used to describe red wine aroma in all three cultures (Fig. 2b and Supplementary figure S4). Berry attributes were used by the South African cohort, and red fruit in the French and Portuguese. South African and French consumers also used spicy and undergrowth to describe the *Red wine* concept. South Africans used attributes such as heady/ rich, deep, heavy, smoke, and black fruit to describe the *Red Wine* concept generally absent from other cultures. Body and dried fruit attributes were associated with the Portuguese *Red wine* concept.

## Discussion

Consumers use different descriptive words to draw a mental picture of wine aroma. These pictures are shaped by personal experiences, but everyone’s experiences are different. Therefore, we collected data from three countries with different winemaking histories to evaluate the degree to which personal experience is impacted by cultural background in terms of the mental pictures describing the aromas of global, *Red*, and *White wine* concepts (Figs. 1 and 2). The Douro region in Portugal, grows several native cultivars, such as Touriga Nacional, Touriga Franca, Tinta Barroca, Tinto Cao and Tinta Roriz (Tempranillo), not commonly found in France and South Africa. In Burgundy, France, only Chardonnay and Pinot noir are cultivated and in the Western Cape, South Africa, Chenin blanc, Sauvignon blanc, Chardonnay, Pinotage, Cabernet Sauvignon, Syrah are commonly grown. In France and Portugal data were collected in specific wine regions with specific cultivars which may also have influenced participants’ wine concepts. Previous work showed that in France, wine producing regions have such strong and distinct identities, that it impacts consumers’ conceptualizations of wine^7^, it is unclear to what extent this regional identity is seen among Portuguese and South African wine consumers.

Our first aim was to evaluate whether wine consumers have different mental representations of *Red* and *White wine* compared to a *Wine* concept. In all three cultural contexts, the data shows that the *Red* and *White wine* concepts are distinct from each other, with the *Wine* concept sharing elements of both. Interestingly, although participants were asked to describe the wine aroma concepts as if to an alien, the elicited attributes included descriptions of taste, mouthfeel, appearance, hedonic characteristics, emotions, intensity, as well as concepts such as quality, minerality and complexity. This suggests that wine aroma is either inextricably linked to these other traits or that wine’s aromatic conceptualization is difficult to describe and that wine consumers do not have the required vocabulary^24,25^. Other research has shown that when wine colour is altered our perception of the wines attributes also changed to align with wine colour rather than with its inherent traits^26–28^. This suggests the existence of an underlying conceptualization used to describe wine based on visual cues, and when the visual cue is removed so is the conceptualization switch^29^. Our data, for the first time, confirms the existence of these conceptual wine representations that underpin the perceptual differences between red and white wines.

Moreover, as seen previously, South Africans favoured a “technical” approach when describing wine aroma by using more specific attributes, whereas French consumers used more generic terms possibly reflecting the “terroir”^9^. Interestingly, only French consumers used a hedonic trait (pleasant, global wine aroma) above the 10% threshold. This suggests a stronger emotional motivation in the French cohort associated with wine consumption^11^. Interestingly, ChatGPT 3.5 was able to accurately identify the *Red* and *White Wine* concepts in all three cultures, affirming that the data collected indeed matches the characteristics associated with these two broad wine styles.

Our second goal was to assess how culture influences these mental representations. This study focuses solely on experiential conceptualizations from consumers, disregarding grape variety. Moreover, the results indicate that expectations are comparable for the *Wine* and *White wine* concepts, demonstrated by their strong correlation across cultures. Indeed, the aroma data also shows that agreement seen across cultures, largely lies in the use of generic aroma attributes but as the information become more precise linguistic, cultural, and wine concept differences emerge.

South African, and Portuguese wine consumers share an association of freshness (acid, crisp) in their conceptualization of white wine, largely absent from the French data. This is possibly due to differences in wine style. Many wines in Burgundy undergo malolactic fermentation (MLF) which leads to acidity reduction, possibly explaining the less frequent association with acidity. Red wines are generally considered more complex than white wine, in terms of aroma and flavour^30^. It is possible that this complexity in addition to different wine styles and cultivars contributes to differences observed between the three participating countries. Portuguese consumers cited body, and boldness/intensity as being essential to their *Red wine* conceptualization. The French *Red wine* concept was described as having animal and red fruit character with the presence of tannins. Whereas, South African’s have cited an association of spicy and smoke with their *Red wine* concept. It is intriguing that the South African conception of wine aligns more closely with that of the Portuguese consumers rather than French, despite both South Africa and France cultivating the same grape varieties.

Research indicates that experts often form idealized prototypes of various wine styles based on their personal experiences, serving as benchmarks against which all wines are evaluated^15,19,31–33^. Our data suggests that wine consumers may similarly rely on these preexisting wine prototypes to articulate their conception of wine. This influence is evident in the cultural differences seen in the *Red wine* concept, also highlighting the opportunity to define and differentiate the *White wine* prototypes within each cultural context more clearly.

## Conclusion

The difficulty consumers had in describing wine aroma serves as a valuable reminder of the importance of fully engaging our senses as we experience life. This prompts us to delve deeper into the meanings we attach to the words we use to describe wine.

The study has several limitations that should be acknowledged. First, the sample size was relatively small, which restricts our ability to perform detailed segmentation based on socio-demographic data or specific wine consumer behaviours. Consequently, the findings may not be fully representative of the broader population, although the stability of the South African data from a larger sample is promising (Supplementary figure S1). Additionally, consumers perceive food products wholistically, so it may be unnatural for them to split their perception into different sensory modalities. This might impact their mental constructs of these individual modalities (odour, taste, mouthfeel).

Nonetheless, the data shows the existence of *Red, White* and global *Wine* conceptualizations. These conceptualizations are established through personal experience. Particularly interesting is the discovery that while the conceptualizations of white wine and generic wine aromas are shared across cultures, the conceptualization of red wine aroma varies. This study enriches our understanding of consumer expectations across diverse contexts and underscores the potential for targeted marketing strategies for red wine. Future research could delve into how and if these conceptualizations evolve with training, further explore regional differences within countries and could intentionally explore facets of these wine conceptualizations that extend beyond aroma.

### Supplementary Information

Supplementary Figures.

## Data Availability

The data collected and analysed during this study are available from the corresponding author on reasonable request.

## References

[1] McGovern PE (2003). Ancient Wine : The Search for the Origins of Viniculture.

[2] Valente CC, Bauer FF, Venter F, Watson B, Nieuwoudt HH (2018). Modelling the sensory space of varietal wines: Mining of large, unstructured text data and visualisation of style patterns. Sci. Rep..

[3] Fischler C (1988). Food, self and identity. Soc. Sci. Inform. Sur les Sci. Soc..

[4] Lo Monaco G, Bonetto E (2019). Social representations and culture in food studies. Food Res. Int..

[5] Weightman C, Bauer FF, Terblanche NS, Valentin D, Nieuwoudt HH (2019). An exploratory study of urban South African consumers’ perceptions of wine and wine consumption: Focus on social, emotional, and functional factors. J. Wine Res..

[6] Silva AP (2016). Functional or emotional? How Dutch and Portuguese conceptualise beer, wine and non-alcoholic beer consumption. Food Qual. Prefer..

[7] Rodrigues H, Parr WV (2019). Contribution of cross-cultural studies to understanding wine appreciation: A review. Food Res. Int..

[8] Country Statistics|OIV. *International Organisation of Vine and Wine* https://www.oiv.int/what-we-do/country-report? (2020).

[9] Valentin D (2021). The impact of “Wine Country of Origin” on the perception of wines by south African and French wine consumers: A cross-cultural comparison. Foods.

[10] Ferreira C, Lourenço-Gomes L, Pinto LMC, Silva AP (2019). Is there a gender effect on wine choice in Portugal?—A qualitative approach. IJWBR.

[11] Do V-B, Patris B, Valentin D (2009). Opinions on wine in a new consumer country: A comparative study of Vietnam and France. J. Wine Res..

[12] Mouret M, Lo Monaco G, Urdapilleta I, Parr WV (2013). Social representations of wine and culture: A comparison between France and New Zealand. Food Qual. Prefer..

[13] Barbe J-C, Garbay J, Tempère S (2021). The sensory space of wines: From concept to evaluation and description. A review. Foods.

[14] Fairbairn SC, Monforte AR, Brand J, Silva Ferreira AC, Bauer F (2022). Yeast metabolic activity is sufficient to create a wine like aromatic feature in a synthetic grape must—a sensory-driven approach. OENO One.

[15] Parr WV, Mouret M, Blackmore S, Pelquest-Hunt T, Urdapilleta I (2011). Representation of complexity in wine: Influence of expertise. Food Qual. Prefer..

[16] Jose-Coutinho A, Avila P, Ricardo-Da-Silva JM (2015). Sensory profile of Portuguese white wines using long-term memory: A novel nationwide approach: Sensory profile of Portuguese white wines. J. Sens. Stud..

[17] Otheguy M, Honoré-Chedozeau C, Valentin D (2021). Do wine experts share the same mental representation? A drawing elicitation study with wine makers, sellers, and critics. Food Qual. Prefer..

[18] Sáenz-Navajas MP (2021). Access to wine experts’ long-term memory to decipher an ill-defined sensory concept: The case of green red wine. OENO One.

[19] Brochet F, Dubourdieu D (2001). Wine descriptive language supports cognitive specificity of chemical senses. Brain Lang..

[20] Zaichkowsky JL (1994). The personal involvement inventory: Reduction, revision, and application to advertising. J. Advert..

[21] Zaichkowsky JL (1985). Measuring the involvement construct. J. Consum. Res..

[22] O’Cass A (2000). An assessment of consumers product, purchase decision, advertising and consumption involvement in fashion clothing. J. Econ. Psychol..

[23] Noble AC (1987). Modification of a standardized system of wine aroma terminology. Am. J. Enol. Vitic..

[24] Solomon GEA (1997). Conceptual change and wine expertise. J. Learn. Sci..

[25] Speed LJ, Majid A (2020). Grounding language in the neglected senses of touch, taste, and smell. Cognit. Neuropsychol..

[26] Morrot G, Brochet F, Dubourdieu D (2001). The color of odors. Brain Lang..

[27] Parr WV, Geoffrey White K, Heatherbell DA (2003). The nose knows: Influence of colour on perception of wine aroma. J. Wine Res..

[28] Wang QJ, Spence C (2019). Drinking through rosé-coloured glasses: Influence of wine colour on the perception of aroma and flavour in wine experts and novices. Food Res. Int..

[29] Ballester J, Abdi H, Langlois J, Peyron D, Valentin D (2009). The odor of colors: Can wine experts and novices distinguish the odors of white, red, and rosé wines?. Chem. Percept..

[30] Wang QJ, Spence C (2018). Wine complexity: An empirical investigation. Food Qual. Prefer..

[31] Ballester J, Patris B, Symoneaux R, Valentin D (2008). Conceptual vs. perceptual wine spaces: Does expertise matter?. Food Qual. Prefer..

[32] Parr WV (2019). Demystifying wine tasting: Cognitive psychology’s contribution. Food Res. Int..

[33] Parr WV, Green JA, White KG, Sherlock RR (2007). The distinctive flavour of New Zealand Sauvignon blanc: Sensory characterisation by wine professionals. Food Qual. Prefer..

