# Supplementary material for: Cultural differences in wine conceptualization among consumers in France, Portugal and South Africa
Source: Sci Rep. 2024 Jul 10;14:15977. doi: 10.1038/s41598-024-66636-3 (PMC11237039; doi:10.1038/s41598-024-66636-3)
Supplement: Supplementary file 1 — Supplementary Figures. [file 41598_2024_66636_MOESM1_ESM.pdf]

Title: *Cultural differences in wine conceptualization among consumers in France, Portugal and South Africa*

Supplementary material

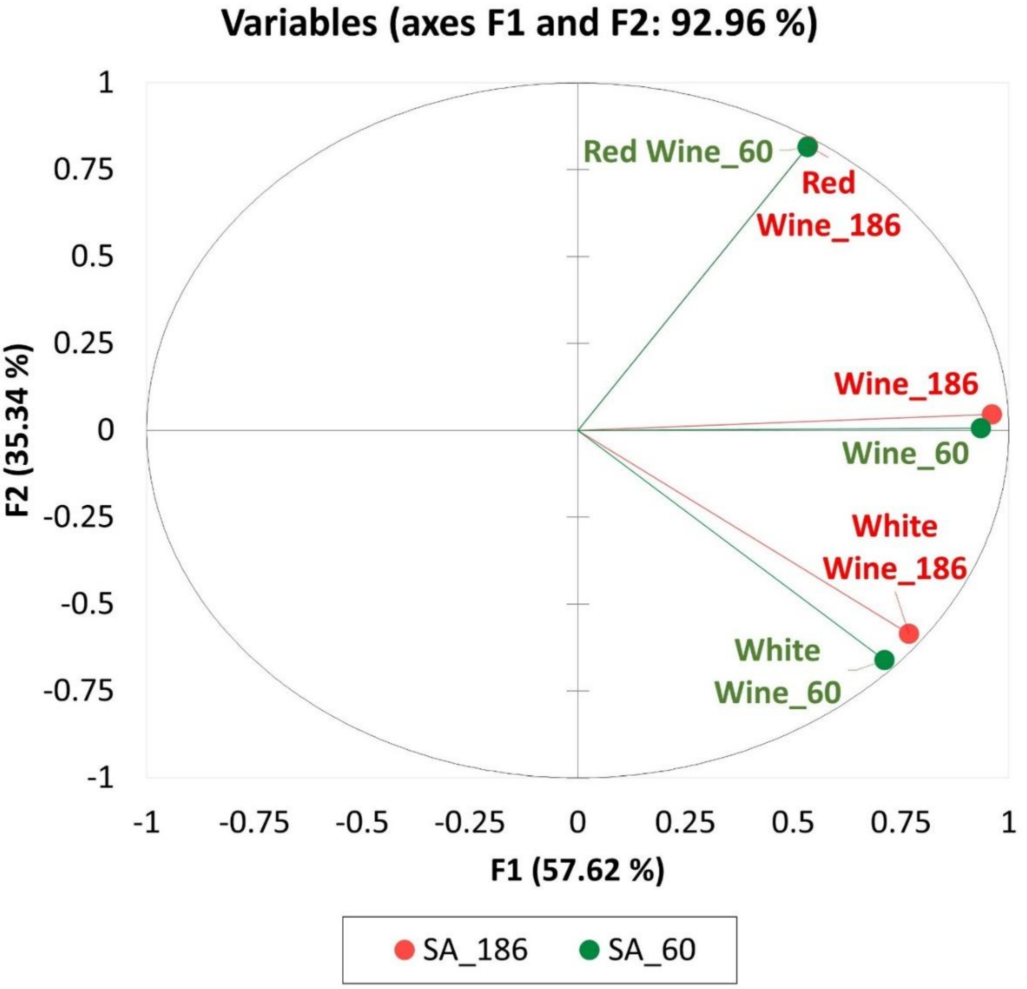

Supplementary figure S1. Multiple correspondence analysis variable map comparing entire South African dataset of 186 to a subset of 60 (Rv coefficient = 0.967). The attributes associated with Wine, Red wine and White wine cited by a minimum of 10% of the participants were used.

A.

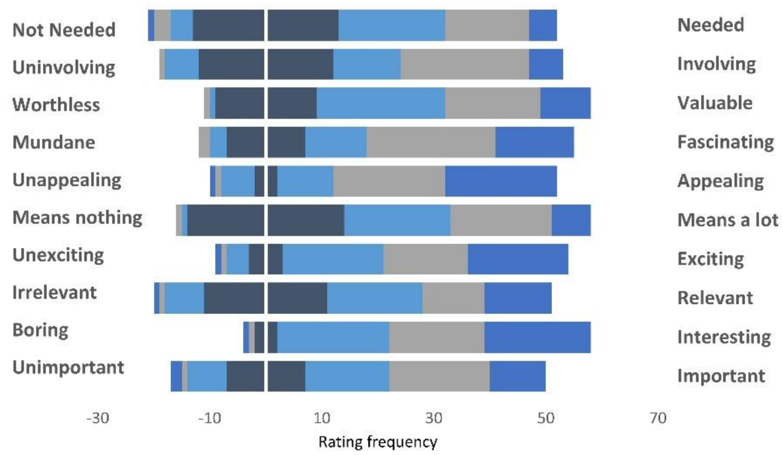

B.

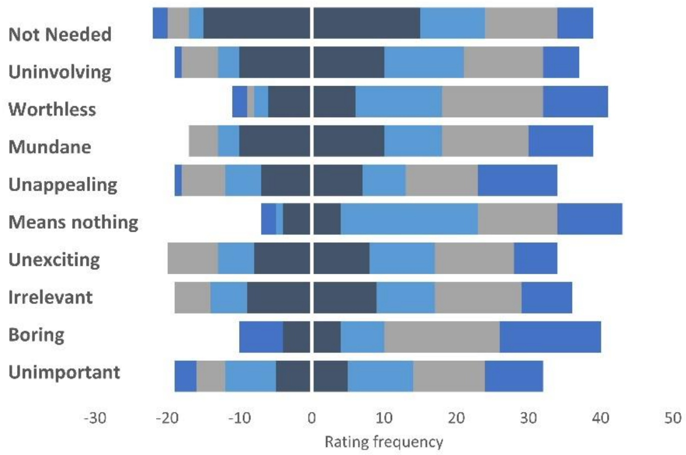

C.

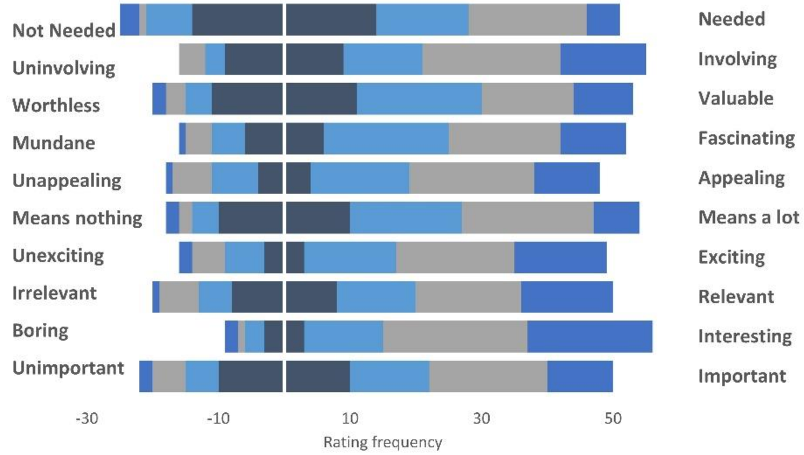

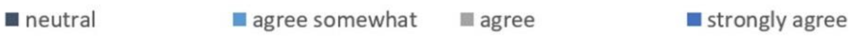

Supplementary figure S2. Frequency distributions summarizing how participants describe their relationship to wine for participants in South Africa (A), France (B) and Portugal (C). Consumers rated whether wine was important/unimportant, interesting/boring, relevant/irrelevant, exciting/unexciting, means a lot/means nothing, appealing/unappealing, fascinating/fascinating, valuable/worthless, involving/uninvolving, and needed/not needed using a 7-point scale: Agree strongly (7), agree (6), agree somewhat (5), neutral (4), agree somewhat (3), agree (2), and agree strongly (1)

.

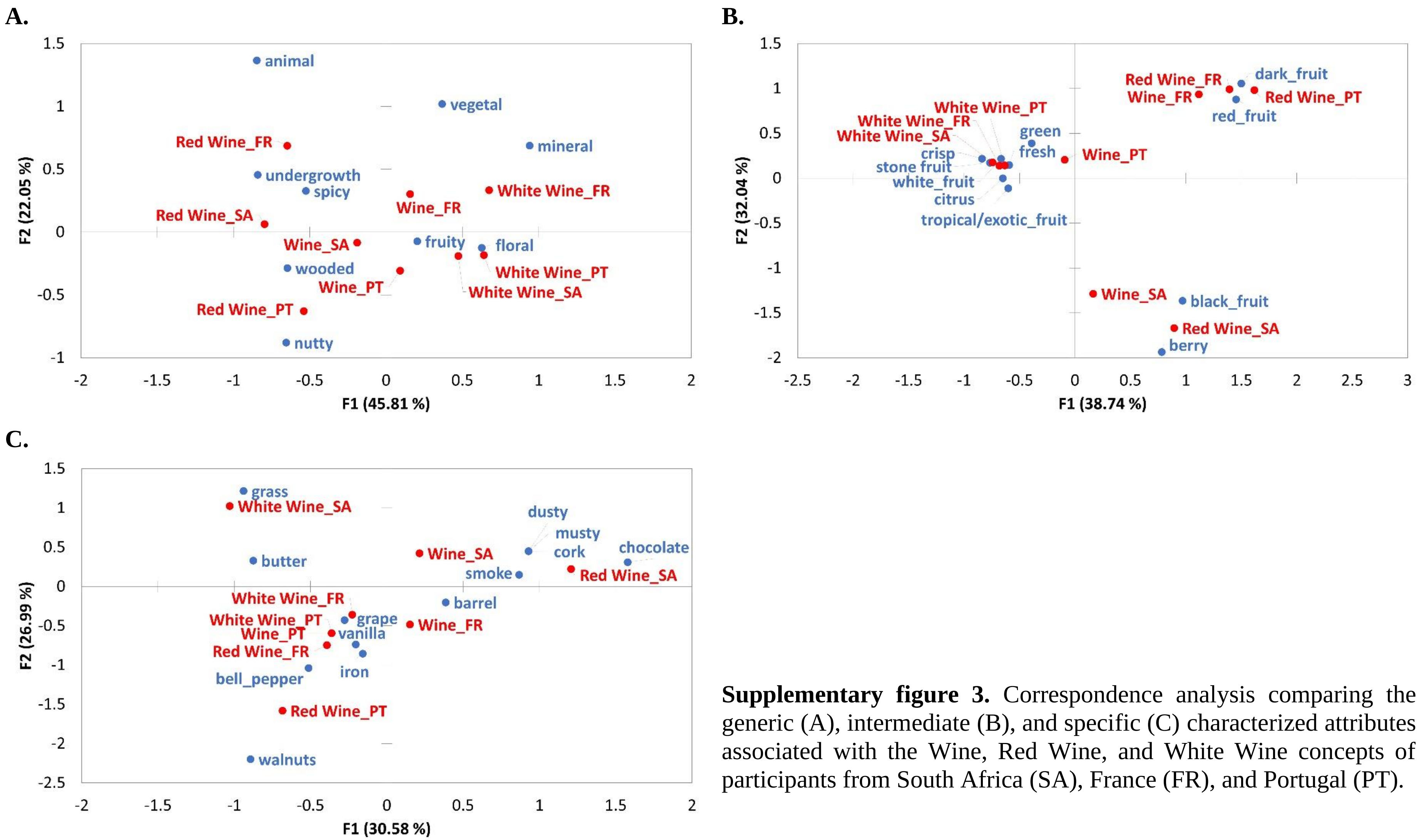

Supplementary figure 3. Correspondence analysis comparing the generic (A), intermediate (B), and specific (C) characterized attributes associated with the Wine, Red Wine, and White Wine concepts of participants from South Africa (SA), France (FR), and Portugal (PT).

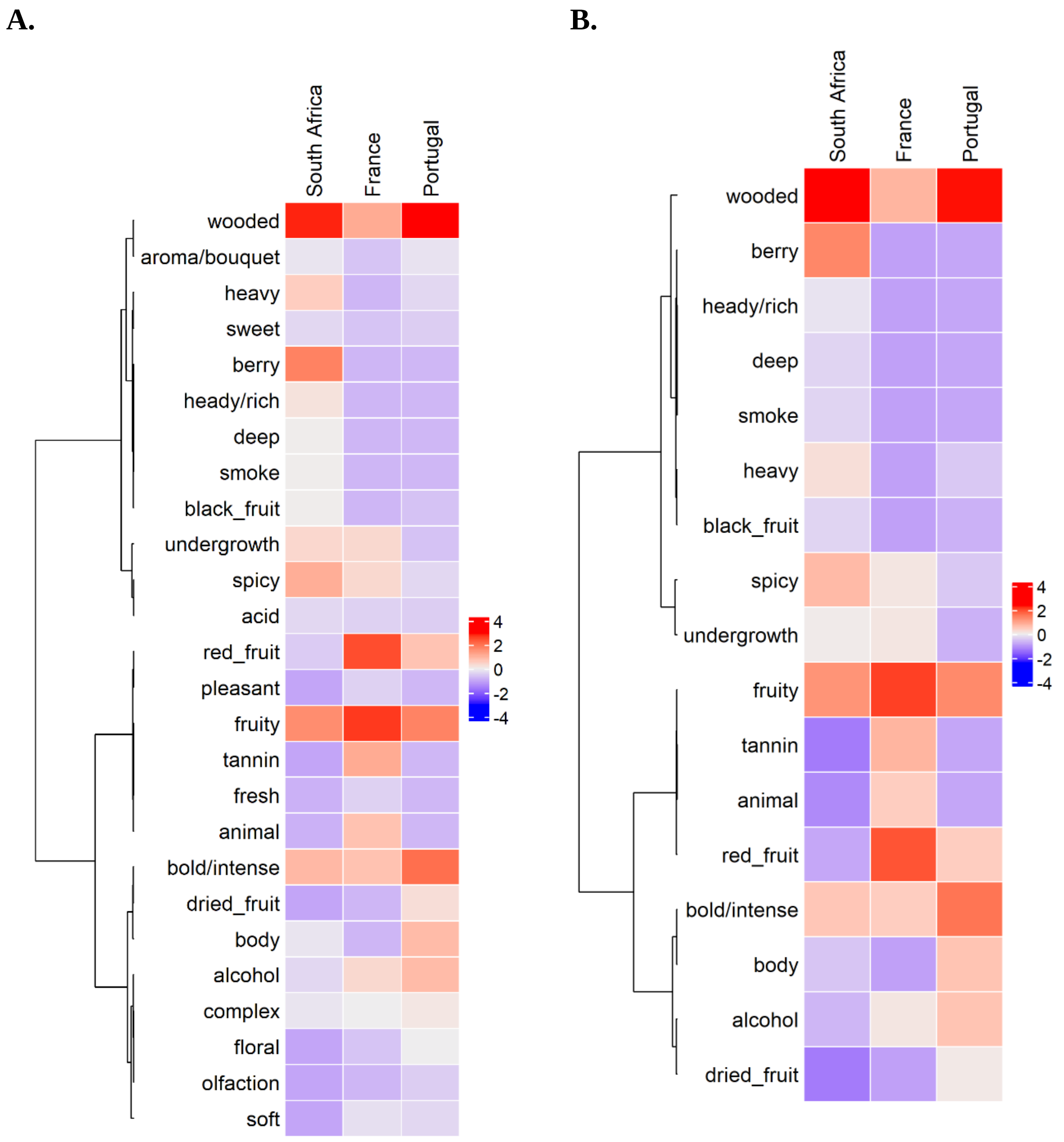

Supplementary figure 4. A summary of the frequency with which South African, French, and Portuguese wine consumers used attributes to describe their mental construct *Red Wine* (RW). The characterized data used was cited by all (A) or at least 10% (B) of the participants in at least one country. Cluster analysis was also performed, using the Ward method to determine the linkage between treatments, and Pearson's correlation coefficient was used as the distance measure. The frequency data were auto-scaled and the heatmap was constructed using the package Complex Heatmap in Rstudio.
